# Supplementary material for: Uphill energy transfer mechanism for photosynthesis in an Antarctic alga
Source: Nat Commun. 2023 Feb 15;14:730. doi: 10.1038/s41467-023-36245-1 (PMC9931709; doi:10.1038/s41467-023-36245-1)
Supplement: Supplementary file 1 — Supplementary Information [file 41467_2023_36245_MOESM1_ESM.pdf]

Supplementary Information

Supplementary Table 1| Statistics of single-particle cryo-EM data and atomic model refinement

	P. crispa frLHC
Data collection	
Microscope	FEI Talos Arctica
Voltage [kV]	200
Detector	Falcon 3EC
Magnification	92 k
Pixel size [Å/pixel]	1.13
Automation software	EPU
Total exposure [e-/Å2]	50
Exposure rate [e-/Å2 frame]	1.00
Number of frames	50
Defocus range [µm]	1.0 to 3.0
Micrographs	1,555
Number of particles for Class2D	696,095
Number of particles for Class3D	654,477
Number of particles for Refine3D	99,510
Symmetry imposed	C11
Map resolution [Å]<br>FSC threshold	3.13<br>0.143
Map resolution range [Å]	2.93 – 12.06
Model refinement	
Program	*phenix.real_space_refine*
Resolution limit	3.13
Number of chains	11
Number of residues	2,651
Number of nonhydrogen atoms	29,271
RMS bond length	0.014
RMS bond angle	1.598
Ramachandran plot	
Preferred [%]	88.70
Allowed [%]	11.30
Outliers [%]	0.00
MolProbity score	
Clash score	14.73
Rotamer outliers [%]	6.74
Overall score	2.89
EMDB/PDB codes	EMD-35080/8HW1

Supplementary Table 2| Ligands of Chls in the structural model of Pc-frLHC

**Chl**	**Ligand**	**Mg–ligand distance (Å)**
601	Unidentified*	
602	Glu124	2.77
603	Asn127	2.56
604	Unidentified*	
609	Glu178	2.23
610	Glu219	3.58
611	Glu63	2.62
612	Asn222	3.36
613	Gln236	2.67
614	His251	3.48
708	His171	2.59

*No protein ligands were found for Chl601 or 604. Water molecules may coordinate the Mg ions of these Chls.

Supplementary Table 3| Exciton couplings (cm^{-1}) between Chl *a* molecules bound to two subunits. Strong interactions are highlighted with hatching.

	601	602	603	604	609	610	611	612	613	614	708
**601**	-	75	1	-2	4	-5	-5	-6	-13	-21	-2
**602**	75	-	34	8	-39	-11	-5	-8	-2	2	9
**603**	1	34	-	-4	154	15	-1	8	4	0	-67
**604**	-2	8	-4	-	-6	-3	-4	-2	3	-1	1
**609**	4	-39	154	-6	-	3	6	0	-4	-1	86
**610**	-5	-11	15	-3	3	-	-36	-86	8	4	-6
**611**	-5	-5	-1	-4	6	-36	-	-73	-7	-7	-2
**612**	-6	-8	8	-2	0	-86	-73	-	25	14	-1
**613**	-13	-2	4	3	-4	8	-7	25	-	66	3
**614**	-21	2	0	-1	-1	4	-7	14	66	-	0
**708**	-2	9	-67	1	86	-6	-2	-1	3	0	-
**601'**	2	1	15	1	-11	3	0	1	-2	-1	-15
**602'**	1	-3	5	-2	-2	2	1	0	-1	0	-9
**603'**	0	1	-1	1	1	0	0	0	0	0	0
**604'**	0	0	-1	1	1	0	0	0	0	0	2
**609'**	0	-1	2	-1	-2	1	0	0	0	0	-1
**610'**	0	2	-4	2	7	-2	-1	-1	0	0	3
**611'**	-2	9	-33	6	65	-6	-2	-1	1	1	64
**612'**	0	4	-9	5	22	-1	-1	0	0	0	14
**613'**	-1	2	-9	3	9	-2	-1	-1	2	1	-7
**614'**	-2	-3	-14	-1	15	-2	0	-2	3	0	-92
**708'**	0	0	-1	0	0	0	0	0	0	0	1

Supplementary Table 4| The transition dipole moment (in debye) of the lower (μ_-) and higher (μ_+) mixed excited states of the strongly interacting Chl pairs (hatched in Supplementary Table 3). For simplicity, it was assumed here that both Chls of the pair have the same site energies, which are the excitation energies of the pigments reflecting the effects of their local protein environment except for those of the exciton coupling.

	μ_x	μ_y	μ_z		μ_x	μ_y	μ_z	$\lvert\mu_-\rvert$	$\lvert\mu_+\rvert$
**Chl601**	2.72	−1.32	−3.47	**Chl602**	−3.78	−2.01	−1.67	4.8	4.4
**Chl603**	4.35	0.40	−1.45	**Chl609**	−4.19	−1.90	0.02	6.3	1.5
**Chl603**	4.35	0.40	−1.45	**Chl708**	0.85	−0.37	4.51	4.3	4.9
**Chl609**	−4.19	−1.90	0.02	**Chl708**	0.85	−0.37	4.51	4.9	4.3
**Chl610**	3.53	2.57	−1.45	**Chl612**	4.28	−1.26	−1.13	5.9	2.8
**Chl611**	4.43	0.83	0.93	**Chl612**	4.28	−1.26	−1.13	6.2	2.1
**Chl613**	−0.85	3.16	3.24	**Chl614**	−3.60	2.86	−0.11	3.1	5.7
**Chl609**	-4.19	−1.90	0.02	**Chl611'**	3.28	3.09	0.93	6.4	1.3
**Chl708**	0.85	−0.37	4.51	**Chl611'**	3.28	3.09	0.93	3.9	5.2
**Chl708**	0.85	-0.37	4.51	**Chl614'**	−4.58	0.47	−0.11	4.1	5.1

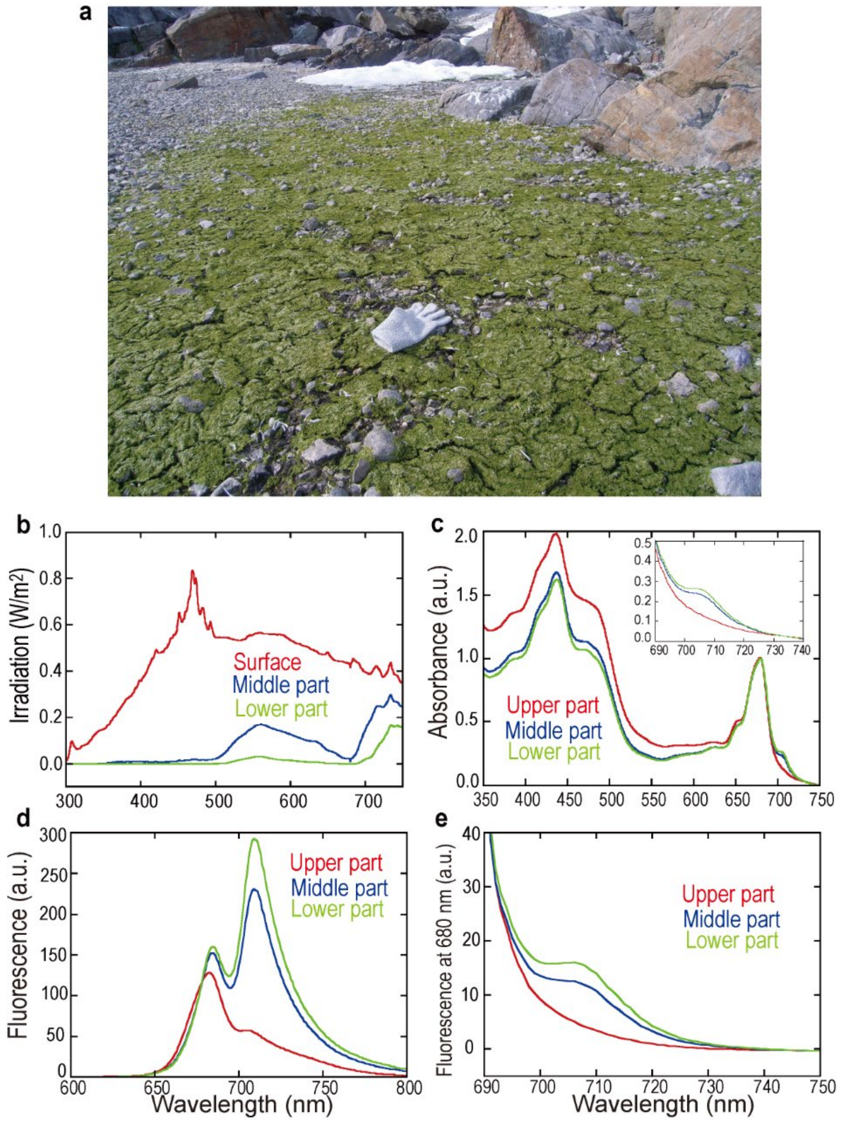

Supplementary Figure 1| *Prasiola crispa* in Antarctica, light conditions of the *P. crispa* colony, and spectroscopic properties of the cells. a: Colonies of *P. crispa* spreading in an Antarctic habitat. **b:** Light spectral conditions on the surface and in the middle and lower parts of a colony of *P. crispa* simulated using an HAL-100 solar simulator (Asahi Spectra, Tokyo, Japan) were measured by a spectrometer, Black comet (StellarNet, Tampa, FL, USA). A drought colony harvested from Antarctica was sliced in the upper, middle, and lower parts. Each part of a colony was sandwiched with quartz plates and vertically set under the irradiation of the solar simulator. The transmitted light of samples was determined by a spectrometer six times under each condition and averaged. **c:** Absorption spectra of thylakoid membranes isolated from the upper, middle, and lower parts of a colony were measured by an MPS-2450 spectrometer (Shimadzu). All spectra were normalized at 679 nm. **d:** Emission fluorescence spectra of thylakoid membranes were measured at room temperature with excitation at 435 nm by an RF-6000 fluorescence spectrometer (Shimadzu). The slit widths of the excitation and emission sides were 5 nm and 3 nm, respectively. **e:** Excitation spectra of fluorescence emission at 680 nm were measured by an RF-6000. The second-order diffraction of excitation light shorter than 400 nm was cut by long-pass filters. The slit widths of the excitation and emission sides were 3 nm and 5 nm, respectively. a.u.: arbitrary unit. Source data are provided as a Source Data file.

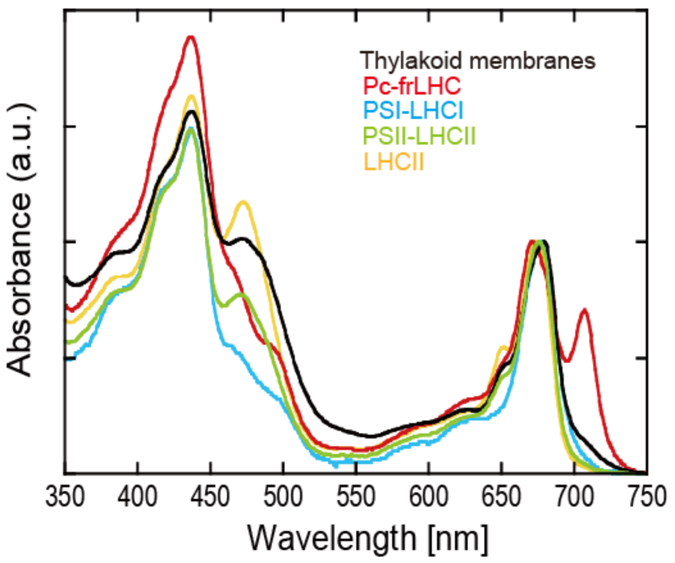

Supplementary Figure 2| Absorbance spectra of isolated proteins. Absorbance spectra of thylakoid membranes (black) and purified fractions of Pc-frLHC (red), PSI-LHCI (blue), PSII-LHCII (green), and LHCII (yellow) were normalized at the peak wavelength of the red absorption band. a.u.: arbitrary unit. Source data are provided as a Source Data file.

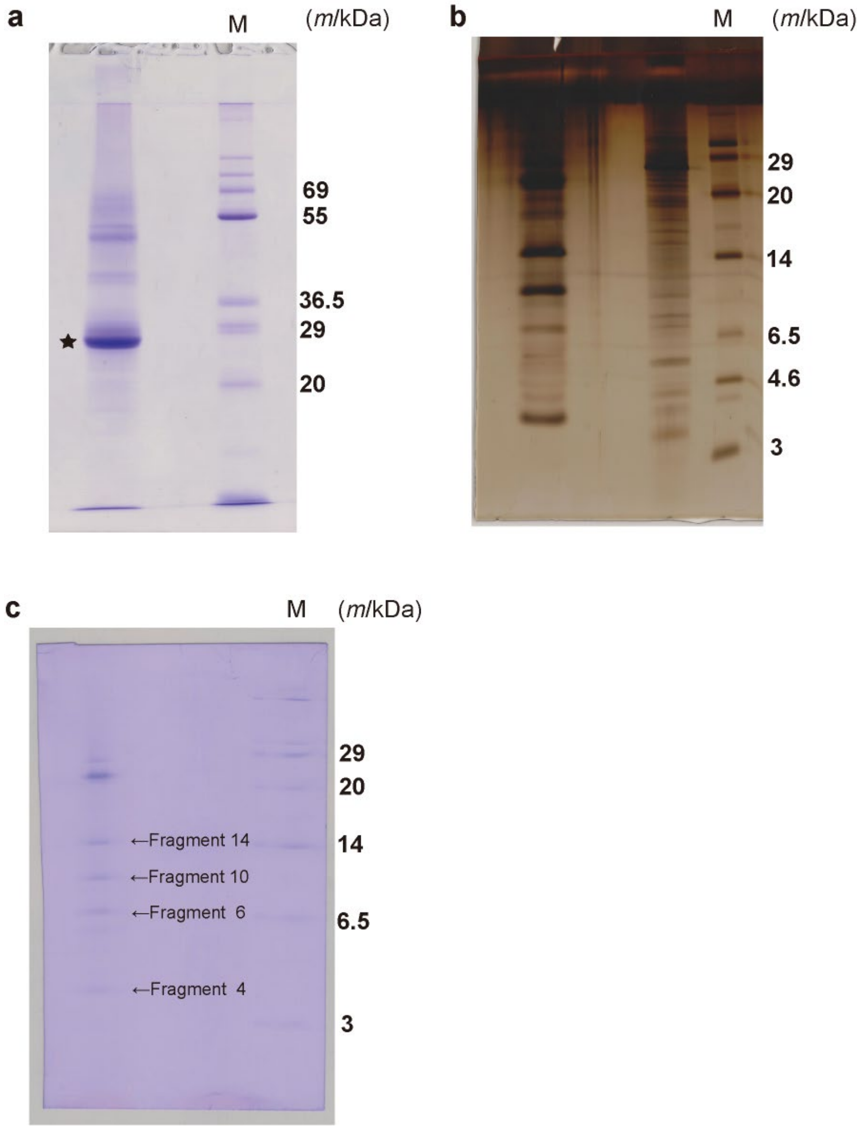

Supplementary Figure 3| Amino acid sequence analysis. a: Pc-frLHC fraction after the DE52 column was loaded onto an SDS-PAGE gel and the separated proteins were stained by CBB. The main protein band detected at 29 kDa (*) was cut out and used for lysyl endopeptidase treatment. The fragmented peptides after lysyl endopeptidase treatment were separated by SDS-PAGE and detected by silver staining (**b**) or electrophoretically blotted onto a PVDF membrane (**c**). **b:** The right and left lanes ae thylakoid membranes and the fragmented peptides of the 29 kDa protein of (**a**), respectively. **c:** N-terminal amino acid sequences of fragmented peptides of 14, 10, 6, and 4 kDa were determined by a Procise 492cLC peptide sequencer (Applied Biosystems). Fragments 14 and 10 had the same N-terminal sequence. The data of **a** and **b** were representative of two independent experiments. Transfer the fragmented peptides to a PVDF membrane (**c**) was performed only one time and it was used for the amino acid sequence analysis. Source data are provided as a Source Data file.

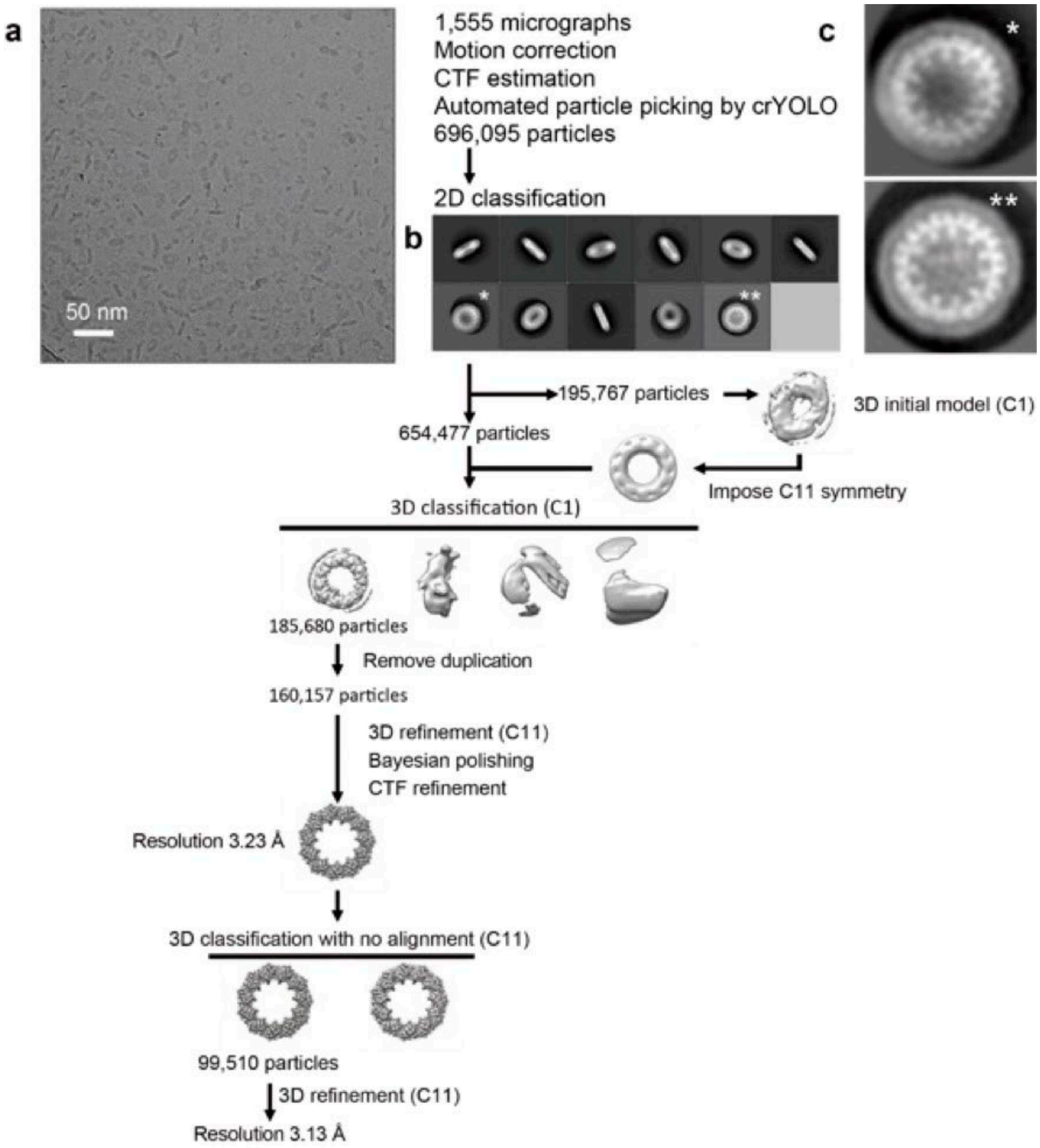

Supplementary Figure 4| Cryo-EM data processing workflow. a: Representative micrograph. **b:** 2D averages of the particle images. **c:** Magnification of two top views (* and **) showing a clear indication of 11 subunits with 11-fold rotational symmetry.

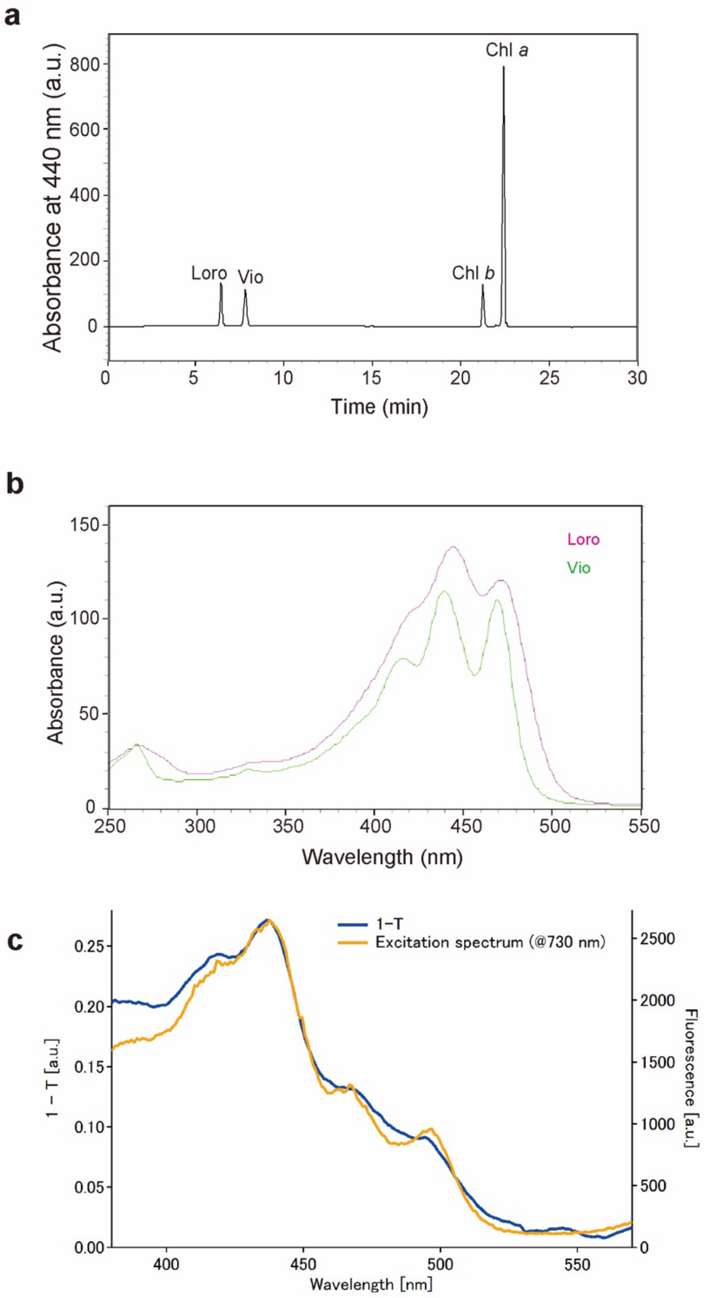

Supplementary Figure 5| Identification of pigments binding to Pc-frLHC with high-performance liquid chromatography. Pigment analysis was performed with HPLC using a C18-column as described in our most recent article[45]. The purified Pc-frLHC fraction was injected directly into the HPLC system and the absorbance at 400 nm was monitored. Loroxanthin (loro), violaxanthin (vio), chlorophyll *b* (Chl *b*), and chlorophyll *a* (Chl *a*) were identified from the retention time (**a**) and absorbance spectra (**b**). Loroxanthin was also identified by its molecular weight (584) using liquid chromatography–mass spectrometry. **c,** Comparison of the excitation spectrum monitored at 730 nm to the 1-transmittance spectrum. Both spectra were measured at 77 K. Source data are provided as a Source Data file.

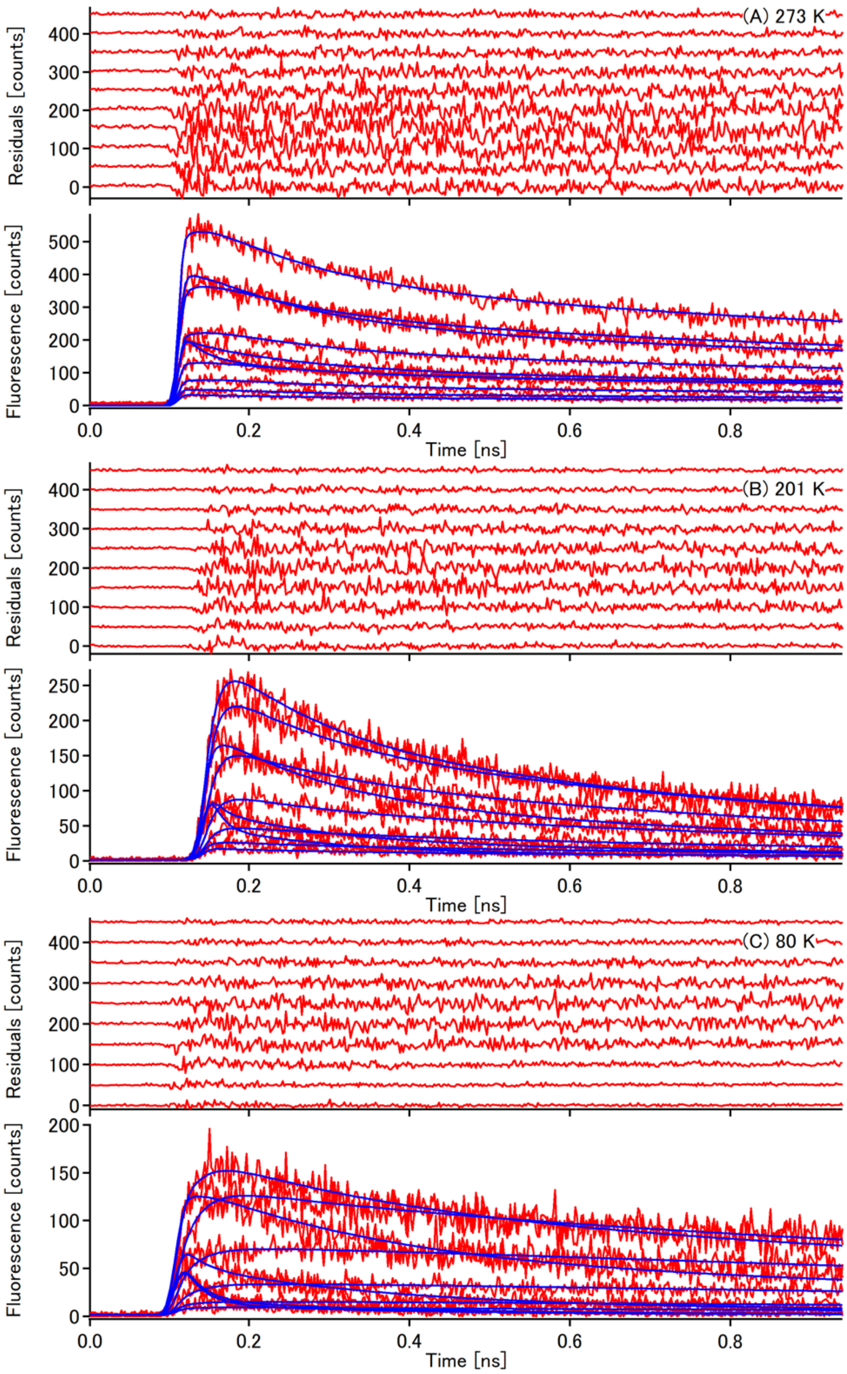

Supplementary Figure 6| Comparison of the experimental (red) and model (blue) fluorescence decays at 273 K (A), 201 K (B), and 80 K (C). Residuals are shown in the upper panel with vertical offsets. Curves from 680 nm to 770 nm at 10 nm intervals are shown. Source data are provided as a Source Data file.
