# Supplementary material for: Uphill energy transfer mechanism for photosynthesis in an Antarctic alga
Source: Nat Commun. 2023 Feb 15;14:730. doi: 10.1038/s41467-023-36245-1 (PMC9931709; doi:10.1038/s41467-023-36245-1)
Supplement: Supplementary file 3 — Reporting Summary [file 41467_2023_36245_MOESM3_ESM.pdf]

nature portfolio

Corresponding author(s): Makiko Kosugi, Yutaka Shibata, Toshiya Senda

Last updated by author(s): Jan 14, 2023

Reporting Summary

Nature Portfolio wishes to improve the reproducibility of the work that we publish. This form provides structure for consistency and transparency in reporting. For further information on Nature Portfolio policies, see our Editorial Policies and the Editorial Policy Checklist.

Statistics

For all statistical analyses, confirm that the following items are present in the figure legend, table legend, main text, or Methods section.

n/a	Confirmed	
☐	☒	The exact sample size (n) for each experimental group/condition, given as a discrete number and unit of measurement
☐	☒	A statement on whether measurements were taken from distinct samples or whether the same sample was measured repeatedly
☒	☐	The statistical test(s) used AND whether they are one- or two-sided<br>*Only common tests should be described solely by name; describe more complex techniques in the Methods section.*
☒	☐	A description of all covariates tested
☒	☐	A description of any assumptions or corrections, such as tests of normality and adjustment for multiple comparisons
☒	☐	A full description of the statistical parameters including central tendency (e.g. means) or other basic estimates (e.g. regression coefficient) AND variation (e.g. standard deviation) or associated estimates of uncertainty (e.g. confidence intervals)
☒	☐	For null hypothesis testing, the test statistic (e.g. F, t, r) with confidence intervals, effect sizes, degrees of freedom and P value noted<br>*Give P values as exact values whenever suitable.*
☒	☐	For Bayesian analysis, information on the choice of priors and Markov chain Monte Carlo settings
☒	☐	For hierarchical and complex designs, identification of the appropriate level for tests and full reporting of outcomes
☒	☐	Estimates of effect sizes (e.g. Cohen's d, Pearson's r), indicating how they were calculated

Our web collection on statistics for biologists contains articles on many of the points above.

Software and code

Policy information about availability of computer code

Data collection	Cryo-EM data collection was performed using a Talos Arctica microscope (Thermo Fisher Scientific) operating at 200 kV in nanoprobe mode using EPU software. Micrograph movies were collected by a 4k × 4k Falcon 3EC direct electron detector (in electron counting mode) at a nominal magnification of 92,000 (1.13 Å/pixel).<br>The cryo-EM data processing was processed using an algorithm implemented on RELION3 (doi: 10.7554/eLife.42166). The nonweighted movie sums were used for Contrast Transfer Function (CTF) estimation with the Gctf program (10.1016/j.jsb.2015.11.003). Particles were picked fully automatically using SPHIRE crYOLO (doi: 10.3791/55448, doi: 10.1038/s42003-019-0437-z). RELION3 was used to perform the subsequent processes: 2D classification, ab initio reconstruction, 3D classification, 3D refinement, CTF refinement, and Bayesian polishing.<br>The gold standard FSC resolution with a 0.143 criterion was used as the global resolution estimation. The local resolution was estimated using an algorithm implemented on RELION3. UCSF Chimera was used for visualization (doi: 10.1002/jcc.20084).

Data analysis	The initial model was built using Map_to_Model in PHENIX software (doi: 10.1038/s41592-018-0173-1, doi: 10.1107/S2059798319011471). The model was manually corrected by Coot (doi: 10.1107/S0907444904019158), followed by Real-space Refinement in PHENIX (doi: 10.1107/S2059798318006551). The model was refined by multiple cycles of manual modifications in Coot and Real-space Refinement in PHENIX. NCS restraints were used for the automatic refinement. The refined model was validated using MolProbity in PHENIX (doi: 10.1002/pro.3330). FSC between the map and the model were calculated by PHENIX. UCSF Chimera and PyMOL (Schrödinger, New York, NY, USA) were used for visualization. The fitting analysis of absorbance spectrum (Fig.1d) with Gaussian functions was performed by Magic plot 2.7.2 (Magicplot Systems, St. Petersburg, Russia). The kinetic analysis of excitation energy transfer in Fig.8 and Supplementary Fig.6 was conducted using a software igor pro ver. 6 (WaveMetrics, Inc. Portland, USA).The signal peptides and the transmembrane helices of Cr_Lhca2, Bc_LhcaJ and Ds_Lhca5 were predicted from the 3D structures registered in the PDB (Cr_Lhca2; 6JO5, 6IJO, Bc_LhcaJ; 6IGZ, Ds_Lhca5; 6SL5) and from the results of secondary structural prediction using Jpred 4 (doi: 10.1093/nar/gkv332) and TargetP-2.0 (doi: 10.26508/lsa.201900429). Evolutionary analyses of LHCs in Fig. 3 were conducted with the neighbor-joining method in MEGA7 (10.1093/molbev/msw054).

For manuscripts utilizing custom algorithms or software that are central to the research but not yet described in published literature, software must be made available to editors and reviewers. We strongly encourage code deposition in a community repository (e.g. GitHub). See the Nature Portfolio guidelines for submitting code & software for further information.

Data

Policy information about availability of data

All manuscripts must include a data availability statement. This statement should provide the following information, where applicable:

- Accession codes, unique identifiers, or web links for publicly available datasets
- A description of any restrictions on data availability
- For clinical datasets or third party data, please ensure that the statement adheres to our policy

The Cryo-EM map of Pc-frLHC is deposited in the Electron Microscopy Data Bank under accession code EMD-35080. Structural coordinates related to the cryo-EM map are deposited at the Protein Data Bank under accession code 8HW1.
The peptide sequence data of Pc-frLHC were deposited in the UniProt Knowledgebase under accession number C0HLU5. The cDNA sequence of the Pc-frLHC gene was submitted to Third PArty data (TPA) of the DDBJ/EMBL/GenBank databases and was assigned the accession number TPA: BR001753.

Human research participants

Policy information about studies involving human research participants and Sex and Gender in Research.

Reporting on sex and gender	Our study did not deal with "sex and gender".
Population characteristics	Our study is not including human research.
Recruitment	Any recruitment was not conducted in our research.
Ethics oversight	Our study was not needed ethics oversight because green algae are not the target of that.

Note that full information on the approval of the study protocol must also be provided in the manuscript.

Field-specific reporting

Please select the one below that is the best fit for your research. If you are not sure, read the appropriate sections before making your selection.

☒ Life sciences ☐ Behavioural & social sciences ☐ Ecological, evolutionary & environmental sciences

For a reference copy of the document with all sections, see nature.com/documents/nr-reporting-summary-flat.pdf

Life sciences study design

All studies must disclose on these points even when the disclosure is negative.

Sample size	We did not perform statistical method to determine the sample size. Sample size of the experiments was set as small as possible because the Prasiola crispa sample harvested from Antarctica was limited. The colony size for the measurement of transmittance spectra in Supplementary Fig. 1b was determined under consideration of the irradiation area and the sensor size. The amount of protein sample used for the biochemical experiments (Figs.1, 8, Supplementary Figs. 1c-e, 2, 3, 5, 6) was modified based on the previous experience to obtain suitable signals.<br>For single particle analysis, sample size was determined by available machine time of the cryo-EM for data collection. 1,555 micrographs were acquired and the number of particles for Class2D, Class3D and Refine3D were 696,095, 654,477 and 99,510, respectively.
Data exclusions	In the process of 2D and 3D classification of single particle analysis, 86% of the total particles were eliminated by the algorithm on RELION3.
Replication	Supplementary Fig.1: The transmitted light of samples were determined by a spectrometer 6 times under each condition and averaged.

We prepared Pc-frLHC sample for cryo-EM analysis three times, and obtained microfigures for single particle analysis at the third time preparation. The biochemical analysis using isolated thylakoids or purified proteins (Figs.1, 8, Supplementary Figs. 1c-e, 2, 3, 5, 6) were performed by using single measurement data because these samples were well homogenized. To confirm the reproducibility, absorbance spectra and fluorescence spectra in Fig. 1b,d and Supplementary Figs. 1c-e, 2 were replicated several times. hrCN-PAGE and SDS-PAGE in Fig.1c were performed two times and confirmed the reproducibility. HPLC analysis in Supplementary Fig.5a was performed three times. Spectroscopic analysis in Fig. 8 were repeated two or three times and confirmed the reproducibility. The data of Supplementary Fig 3a and 3b were representative of two independent experiments. Transfer the peptides to a PVDF membrane of Supplementary Fig 3c was performed only one time and it was used for the amino acid sequence analysis.

Randomization	Prasiola crispa's thalli constructing layered colonies were separated layer by layer and homogenized before use. Thalli used for Pc-frLHC preparation were selected randomly from the homogenized samples.
Blinding	Blinding process was not needed in our experiments, because all of the result of the experiments didn't depend on subjective view.

Reporting for specific materials, systems and methods

We require information from authors about some types of materials, experimental systems and methods used in many studies. Here, indicate whether each material, system or method listed is relevant to your study. If you are not sure if a list item applies to your research, read the appropriate section before selecting a response.

Materials & experimental systems

n/a	Involved in the study
☒	☐ Antibodies
☒	☐ Eukaryotic cell lines
☒	☐ Palaeontology and archaeology
☐	☒ Animals and other organisms
☒	☐ Clinical data
☒	☐ Dual use research of concern

Methods

n/a	Involved in the study
☒	☐ ChIP-seq
☒	☐ Flow cytometry
☒	☐ MRI-based neuroimaging

Animals and other research organisms

Policy information about studies involving animals; ARRIVE guidelines recommended for reporting animal research, and Sex and Gender in Research

Laboratory animals	Our study did not use laboratory animals.
Wild animals	Our study did not use wild animals.
Reporting on sex	Our study is about photosynthesis, so do not relate to sex and gender research.
Field-collected samples	Prasiola crispa cells were harvested from Antarctica under the Antarctic Treaty and the Protocol on Environmental Protection to the Antarctic Treaty. Transportation of the samples to Japan was done in accordance with Plant Protection Act of Japan.
Ethics oversight	Our study was not needed ethics oversight because green algae are not the target of that.

Note that full information on the approval of the study protocol must also be provided in the manuscript.
